# Supplementary material for: Trends in prostate cancer incidence and mortality to monitor control policies in a northeastern Brazilian state
Source: PLoS One. 2021 Mar 25;16(3):e0249009. doi: 10.1371/journal.pone.0249009 (PMC7993820; doi:10.1371/journal.pone.0249009)
Supplement: S1 Table — (PDF) [file pone.0249009.s001.pdf]

S1 Table. Number of incident cases, age-standardized rates and confidence intervals, cancer registry area (CR).

			Absolute numbers																
	Total	Ukn	0	5	10	15	20	25	30	35	40	45	50	55	60	65	70	75	80+
			4	9	14	19	24	29	34	39	44	49	54	59	64	69	74	79	
1996	52	0	0	0	0	1	0	0	0	0	0	3	2	2	7	12	10	15	0
1997	52	0	0	0	0	0	0	0	0	0	0	2	0	2	6	8	9	9	16
1998	89	0	0	0	0	0	0	0	0	0	0	3	5	7	4	15	19	17	19
1999	126	0	0	0	0	0	0	0	0	0	0	2	2	9	14	25	21	23	30
2000	138	2	0	0	0	0	0	0	0	0	0	4	5	6	19	31	29	16	26
2001	126	0	0	0	0	0	0	0	0	0	0	2	6	18	11	24	16	15	34
2002	147	0	0	0	0	0	0	0	0	0	1	2	10	17	19	20	26	26	26
2003	155	1	0	0	0	0	0	0	0	0	0	4	8	13	17	32	21	33	26
2004	189	1	0	0	0	0	0	0	0	0	2	1	8	24	21	23	35	26	48
2005	226	1	0	0	0	0	0	0	0	0	1	4	14	15	32	32	51	36	40
2006	217	0	0	0	0	0	0	0	0	0	1	4	7	17	43	46	35	23	41
2007	252	0	0	0	0	0	0	0	0	0	0	2	12	26	39	51	44	35	43
2008	234	0	0	0	0	0	0	0	0	0	0	6	16	34	50	35	35	28	30
2009	236	0	0	0	0	0	0	0	0	1	1	2	18	29	45	48	41	20	31
2010	232	0	0	0	0	0	0	0	0	0	2	3	17	25	55	47	28	21	34
2011	236	0	0	0	0	0	0	0	0	1	2	12	10	27	40	38	49	30	27
2012	223	7	0	0	0	0	0	0	0	0	2	11	5	23	38	48	35	22	32
2013	255	6	0	0	0	0	0	0	0	0	4	4	16	22	52	55	36	36	24
2014	200	0	0	0	0	0	0	0	0	0	1	1	12	25	39	36	34	20	32
2015	210	0	0	0	0	0	0	0	0	0	3	3	12	28	35	38	43	16	32
	3595																		

	Age-specific rates																		
	0	5	10	15	20	25	30	35	40	45	50	55	60	65	70	75	80+	Crude	Ag sd
	4	9	14	19	24	29	34	39	44	49	54	59	64	69	74	79		rate	rate
1996	0,0	0,0	0,0	4,2	0,0	0,0	0,0	0,0	0,0	32,9	29,8	42,3	202,9	466,6	514,4	1239,7	0,0	26,0	50,3
1997	0,0	0,0	0,0	0,0	0,0	0,0	0,0	0,0	0,0	21,6	0,0	41,7	171,3	306,4	456,2	732,9	1231,7	25,6	47,8
1998	0,0	0,0	0,0	0,0	0,0	0,0	0,0	0,0	0,0	32,0	72,4	144,1	112,8	567,5	951,0	1366,6	1444,9	43,2	80,0
1999	0,0	0,0	0,0	0,0	0,0	0,0	0,0	0,0	0,0	21,1	28,6	183,0	390,0	934,2	1038,1	1826,8	2253,9	60,4	115,2
2000	0,0	0,0	0,0	0,0	0,0	0,0	0,0	0,0	0,0	37,8	59,0	103,1	446,1	1071,2	1324,8	1096,6	1678,5	63,9	113,6
2001	0,0	0,0	0,0	0,0	0,0	0,0	0,0	0,0	0,0	17,5	66,9	289,5	252,1	803,8	717,5	1035,9	2335,2	55,0	98,2
2002	0,0	0,0	0,0	0,0	0,0	0,0	0,0	0,0	6,8	16,8	106,5	255,1	417,9	634,9	1133,9	1760,3	1762,7	62,6	110,6
2003	0,0	0,0	0,0	0,0	0,0	0,0	0,0	0,0	0,0	32,1	81,5	182,2	357,4	970,6	890,2	2192,7	1728,7	64,4	113,7
2004	0,0	0,0	0,0	0,0	0,0	0,0	0,0	0,0	12,7	7,7	78,1	315,8	419,5	669,6	1438,0	1696,0	3137,3	76,8	131,7
2005	0,0	0,0	0,0	0,0	0,0	0,0	0,0	0,0	6,1	29,5	130,9	186,8	602,6	895,4	2023,0	2301,8	2562,5	89,8	156,2
2006	0,0	0,0	0,0	0,0	0,0	0,0	0,0	0,0	5,9	28,4	62,9	201,7	758,1	1219,2	1333,8	1438,4	2572,1	84,5	147,0
2007	0,0	0,0	0,0	0,0	0,0	0,0	0,0	0,0	0,0	13,7	103,6	295,4	643,7	1286,9	1609,4	2149,9	2633,2	96,2	162,2
2008	0,0	0,0	0,0	0,0	0,0	0,0	0,0	0,0	0,0	39,6	132,4	371,1	773,9	842,2	1224,6	1684,7	1791,0	87,7	139,3
2009	0,0	0,0	0,0	0,0	0,0	0,0	0,0	4,9	5,5	12,8	142,7	304,4	656,6	1099,7	1370,8	1175,1	1801,3	86,9	137,1
2010	0,0	0,0	0,0	0,0	0,0	0,0	0,0	0,0	11,0	18,9	130,7	250,2	728,0	943,0	819,7	1053,2	1459,2	87,4	117,3
2011	0,0	0,0	0,0	0,0	0,0	0,0	0,0	4,6	10,6	72,4	73,4	260,3	526,0	770,9	1572,0	1673,2	1511,8	84,1	126,8
2012	0,0	0,0	0,0	0,0	0,0	0,0	0,0	0,0	10,4	65,0	35,6	212,4	476,7	908,4	1084,9	1194,4	1772,9	78,3	112,5
2013	0,0	0,0	0,0	0,0	0,0	0,0	0,0	0,0	20,3	23,2	110,6	194,7	624,3	971,6	1077,5	1895,7	1312,2	88,2	123,7
2014	0,0	0,0	0,0	0,0	0,0	0,0	0,0	0,0	5,0	5,7	79,6	212,4	445,8	604,3	977,9	1013,2	1699,4	68,2	95,8
2015	0,0	0,0	0,0	0,0	0,0	0,0	0,0	0,0	14,6	16,9	77,2	227,8	381,4	604,1	1191,5	782,4	1664,1	70,7	96,5

Year	N	CR	SE	IC95%		ASR	SE	IC95%	
1996	52	26,0	3,598911284	18,89825222	33,00598446	50,33150447	6,979723852	36,65124572	64,01176322
1997	52	25,6	3,545560394	18,61810123	32,51669797	47,77719667	6,625505092	34,79120669	60,76318665
1998	89	43,2	4,581225935	34,2399962	52,19840187	79,97964514	8,477825429	63,3631073	96,59618298
1999	126	60,4	5,383886269	49,8815564	70,98639057	115,2125947	10,26395371	95,09524538	135,3299439
2000	138	63,9	5,441430065	53,25712681	74,58753266	113,5668228	9,667449958	94,61862087	132,5150247
2001	126	55,0	4,898419044	45,38371607	64,58551872	98,22736089	8,75078882	81,0758148	115,378907
2002	147	62,6	5,159300278	52,44096294	72,66542003	110,6096782	9,122932501	92,72873053	128,4906259
2003	155	64,4	5,172608469	54,2601435	74,5367687	113,720511	9,134251249	95,81737851	131,6236434
2004	189	76,8	5,58369498	65,81907255	87,70715688	131,7181959	9,581088942	112,9392616	150,4971303
2005	226	89,8	5,975624313	78,12110769	101,545555	156,2257349	10,3919813	135,8574515	176,5940182
2006	217	84,5	5,736629916	73,26204094	95,74963021	146,9553084	9,975976364	127,4023947	166,508222
2007	252	96,2	6,063137983	84,36558116	108,133082	162,1887969	10,21693386	142,1636065	182,2139873
2008	234	87,7	5,735550959	76,4953789	98,97873866	139,3137365	9,107223859	121,4635777	157,1638953
2009	236	86,9	5,659344813	75,84818886	98,03282053	137,1362565	8,926809947	119,639709	154,632804
2010	232	87,4	5,737274642	76,14250555	98,63262214	117,2656068	7,698864264	102,1758329	132,3553808
2011	236	84,1	5,476733237	73,40077528	94,86956957	126,7973567	8,253804891	110,6198991	142,9748143
2012	223	78,3	5,242730737	68,01491326	88,56641775	112,4878526	7,532743762	97,72367479	127,2520303
2013	255	88,2	5,524915813	77,39699545	99,05466544	123,6736525	7,744744535	108,4939532	138,8533518
2014	200	68,2	4,825481664	58,78467209	77,70056021	95,7640333	6,771539734	82,4918542	109,0362512
2015	210	70,7	4,879070723	61,14147341	80,26743064	96,5427027	6,662079414	83,48502705	109,6003784
	3595					113,7747274	1,897563667	110,0555026	117,4939522
