# Supplementary material for: Trends in prostate cancer incidence and mortality to monitor control policies in a northeastern Brazilian state
Source: PLoS One. 2021 Mar 25;16(3):e0249009. doi: 10.1371/journal.pone.0249009 (PMC7993820; doi:10.1371/journal.pone.0249009)
Supplement: S2 Table — (PDF) [file pone.0249009.s002.pdf]

S2 Table. Number of deaths, age-standardized rates and confidence intervals, cancer registry area (CR).

| | Absolute numbers | | | | | | | | | | | | | | | | | | |
|---|---|---|---|---|---|---|---|---|---|---|---|---|---|---|---|---|---|---|---|
| | Total | Ukn | 0 | 5 | 10 | 15 | 20 | 25 | 30 | 35 | 40 | 45 | 50 | 55 | 60 | 65 | 70 | 75 | 80+ |
| | | | 4 | 9 | 14 | 19 | 24 | 29 | 34 | 39 | 44 | 49 | 54 | 59 | 64 | 69 | 74 | 79 | |
| 1980 | 7 | 0 | 0 | 0 | 0 | 0 | 0 | 0 | 0 | 0 | 0 | 0 | 0 | 0 | 1 | 0 | 1 | 2 | 3 |
| 1981 | 9 | 0 | 0 | 0 | 0 | 0 | 0 | 0 | 0 | 0 | 0 | 0 | 0 | 1 | 0 | 0 | 4 | 1 | 3 |
| 1982 | 12 | 0 | 0 | 0 | 0 | 0 | 0 | 0 | 0 | 0 | 0 | 0 | 1 | 0 | 0 | 1 | 3 | 2 | 5 |
| 1983 | 8 | 0 | 0 | 0 | 0 | 0 | 0 | 0 | 0 | 0 | 0 | 0 | 0 | 0 | 0 | 0 | 3 | 3 | 2 |
| 1984 | 16 | 0 | 0 | 0 | 0 | 0 | 0 | 0 | 0 | 0 | 0 | 0 | 0 | 0 | 2 | 4 | 3 | 3 | 4 |
| 1985 | 14 | 0 | 0 | 0 | 0 | 0 | 0 | 0 | 0 | 0 | 0 | 0 | 0 | 0 | 1 | 2 | 1 | 3 | 7 |
| 1986 | 15 | 2 | 0 | 0 | 0 | 0 | 0 | 0 | 0 | 0 | 1 | 1 | 0 | 0 | 0 | 3 | 3 | 2 | 3 |
| 1987 | 17 | 2 | 0 | 0 | 0 | 0 | 0 | 0 | 0 | 0 | 0 | 0 | 1 | 1 | 4 | 2 | 2 | 4 | 1 |
| 1988 | 14 | 0 | 0 | 0 | 0 | 0 | 0 | 0 | 0 | 0 | 0 | 0 | 0 | 0 | 1 | 5 | 3 | 1 | 4 |
| 1989 | 26 | 0 | 0 | 0 | 0 | 0 | 0 | 0 | 0 | 0 | 0 | 0 | 0 | 2 | 2 | 4 | 5 | 6 | 7 |
| 1990 | 10 | 1 | 0 | 0 | 0 | 0 | 0 | 0 | 0 | 0 | 0 | 0 | 1 | 0 | 0 | 5 | 1 | 1 | 1 |
| 1991 | 8 | 0 | 0 | 0 | 0 | 0 | 0 | 0 | 0 | 0 | 0 | 0 | 0 | 0 | 0 | 2 | 0 | 1 | 5 |
| 1992 | 28 | 1 | 0 | 0 | 0 | 0 | 0 | 0 | 0 | 0 | 0 | 1 | 0 | 2 | 2 | 2 | 8 | 3 | 9 |
| 1993 | 17 | 0 | 0 | 0 | 0 | 0 | 0 | 0 | 0 | 0 | 0 | 0 | 0 | 1 | 3 | 2 | 4 | 4 | 3 |
| 1994 | 26 | 1 | 0 | 0 | 1 | 0 | 0 | 0 | 0 | 0 | 0 | 0 | 0 | 1 | 1 | 2 | 1 | 7 | 12 |
| 1995 | 30 | 2 | 0 | 0 | 0 | 0 | 0 | 0 | 0 | 0 | 0 | 1 | 3 | 2 | 3 | 3 | 4 | 5 | 7 |
| 1996 | 23 | 0 | 0 | 0 | 0 | 0 | 0 | 0 | 0 | 0 | 0 | 1 | 2 | 1 | 0 | 5 | 5 | 6 | 3 |
| 1997 | 18 | 0 | 0 | 0 | 0 | 0 | 0 | 0 | 0 | 0 | 0 | 0 | 0 | 0 | 2 | 2 | 2 | 6 | 6 |
| 1998 | 23 | 0 | 0 | 0 | 0 | 0 | 0 | 0 | 0 | 0 | 0 | 0 | 1 | 1 | 2 | 1 | 4 | 7 | 7 |
| 1999 | 37 | 0 | 0 | 0 | 0 | 0 | 0 | 0 | 0 | 0 | 0 | 1 | 0 | 4 | 0 | 1 | 4 | 9 | 18 |
| 2000 | 37 | 0 | 0 | 0 | 0 | 0 | 0 | 0 | 0 | 0 | 0 | 1 | 1 | 1 | 1 | 6 | 3 | 12 | 12 |
| 2001 | 35 | 0 | 0 | 0 | 0 | 0 | 0 | 0 | 0 | 0 | 0 | 1 | 1 | 2 | 3 | 4 | 4 | 5 | 15 |
| 2002 | 36 | 0 | 0 | 0 | 0 | 0 | 0 | 0 | 0 | 0 | 0 | 0 | 0 | 2 | 2 | 1 | 6 | 6 | 19 |
| 2003 | 32 | 0 | 0 | 0 | 0 | 0 | 0 | 0 | 0 | 0 | 0 | 0 | 3 | 1 | 0 | 1 | 7 | 8 | 12 |
| 2004 | 38 | 0 | 0 | 0 | 0 | 0 | 0 | 0 | 0 | 0 | 0 | 0 | 0 | 3 | 4 | 3 | 6 | 6 | 16 |
| 2005 | 38 | 0 | 0 | 0 | 0 | 0 | 0 | 0 | 0 | 0 | 1 | 0 | 0 | 1 | 5 | 1 | 6 | 6 | 18 |
| 2006 | 46 | 0 | 0 | 0 | 0 | 0 | 0 | 0 | 0 | 0 | 0 | 1 | 0 | 1 | 1 | 7 | 3 | 9 | 24 |
| 2007 | 45 | 0 | 0 | 0 | 0 | 0 | 0 | 0 | 0 | 0 | 0 | 0 | 2 | 2 | 4 | 3 | 10 | 8 | 16 |
| 2008 | 45 | 0 | 0 | 0 | 0 | 0 | 0 | 0 | 0 | 0 | 0 | 0 | 0 | 0 | 8 | 1 | 8 | 7 | 21 |
| 2009 | 41 | 0 | 0 | 0 | 0 | 0 | 0 | 0 | 0 | 1 | 0 | 0 | 0 | 2 | 3 | 2 | 9 | 6 | 18 |
| 2010 | 59 | 0 | 0 | 0 | 0 | 0 | 0 | 0 | 0 | 0 | 0 | 0 | 1 | 3 | 2 | 7 | 12 | 13 | 21 |
| 2011 | 53 | 0 | 0 | 0 | 0 | 0 | 0 | 0 | 0 | 0 | 0 | 1 | 1 | 0 | 3 | 8 | 9 | 6 | 25 |
| 2012 | 47 | 0 | 0 | 0 | 0 | 0 | 0 | 0 | 0 | 0 | 0 | 0 | 1 | 1 | 0 | 2 | 1 | 10 | 32 |
| 2013 | 48 | 0 | 0 | 0 | 0 | 0 | 0 | 0 | 0 | 0 | 1 | 1 | 0 | 1 | 3 | 4 | 6 | 12 | 20 |
| 2014 | 44 | 0 | 0 | 0 | 0 | 0 | 0 | 0 | 0 | 0 | 0 | 0 | 2 | 1 | 4 | 4 | 8 | 4 | 21 |
| 2015 | 55 | 0 | 0 | 0 | 0 | 0 | 0 | 0 | 0 | 0 | 0 | 0 | 2 | 1 | 2 | 10 | 6 | 13 | 21 |
| 2016 | 65 | 0 | 0 | 0 | 0 | 0 | 0 | 0 | 0 | 0 | 0 | 0 | 3 | 3 | 3 | 5 | 8 | 8 | 35 |
| 2017 | 52 | 0 | 0 | 0 | 0 | 0 | 0 | 0 | 0 | 0 | 0 | 0 | 1 | 2 | 4 | 7 | 10 | 14 | 14 |
| 2018 | 44 | 0 | 0 | 0 | 0 | 0 | 0 | 0 | 0 | 0 | 0 | 0 | 0 | 5 | 4 | 4 | 5 | 8 | 18 |
| | 1218 | | | | | | | | | | | | | | | | | | |

| | Age-specific rates | | | | | | | | | | | | | | | | | |
|---|---|---|---|---|---|---|---|---|---|---|---|---|---|---|---|---|---|---|
| | 0 | 5 | 10 | 15 | 20 | 25 | 30 | 35 | 40 | 45 | 50 | 55 | 60 | 65 | 70 | 75 | 80+ | Crude | Ag sd |
| | 4 | 9 | 14 | 19 | 24 | 29 | 34 | 39 | 44 | 49 | 54 | 59 | 64 | 69 | 74 | 79 | | rate | rate |
| 1980 | 0,0 | 0,0 | 0,0 | 0,0 | 0,0 | 0,0 | 0,0 | 0,0 | 0,0 | 0,0 | 0,0 | 0,0 | 43,7 | 0,0 | 88,0 | 285,7 | 574,7 | 5,1 | 12,1 |
| 1981 | 0,0 | 0,0 | 0,0 | 0,0 | 0,0 | 0,0 | 0,0 | 0,0 | 0,0 | 0,0 | 0,0 | 33,7 | 0,0 | 0,0 | 340,7 | 134,0 | 547,4 | 6,3 | 15,0 |
| 1982 | 0,0 | 0,0 | 0,0 | 0,0 | 0,0 | 0,0 | 0,0 | 0,0 | 0,0 | 0,0 | 26,0 | 0,0 | 0,0 | 52,7 | 245,9 | 254,5 | 838,9 | 8,2 | 18,7 |
| 1983 | 0,0 | 0,0 | 0,0 | 0,0 | 0,0 | 0,0 | 0,0 | 0,0 | 0,0 | 0,0 | 0,0 | 0,0 | 0,0 | 0,0 | 237,2 | 362,8 | 310,6 | 5,3 | 11,5 |
| 1984 | 0,0 | 0,0 | 0,0 | 0,0 | 0,0 | 0,0 | 0,0 | 0,0 | 0,0 | 0,0 | 0,0 | 0,0 | 79,0 | 199,2 | 228,8 | 346,0 | 578,9 | 10,2 | 23,0 |
| 1985 | 0,0 | 0,0 | 0,0 | 0,0 | 0,0 | 0,0 | 0,0 | 0,0 | 0,0 | 0,0 | 0,0 | 0,0 | 38,4 | 96,9 | 73,7 | 330,8 | 947,2 | 8,7 | 18,7 |
| 1986 | 0,0 | 0,0 | 0,0 | 0,0 | 0,0 | 0,0 | 0,0 | 0,0 | 12,9 | 17,4 | 0,0 | 0,0 | 0,0 | 141,7 | 214,1 | 211,2 | 381,7 | 9,0 | 16,3 |
| 1987 | 0,0 | 0,0 | 0,0 | 0,0 | 0,0 | 0,0 | 0,0 | 0,0 | 0,0 | 0,0 | 21,2 | 28,9 | 145,7 | 92,1 | 138,3 | 405,7 | 120,2 | 10,0 | 18,8 |
| 1988 | 0,0 | 0,0 | 0,0 | 0,0 | 0,0 | 0,0 | 0,0 | 0,0 | 0,0 | 0,0 | 0,0 | 0,0 | 35,5 | 224,9 | 201,5 | 97,7 | 455,6 | 8,0 | 17,7 |
| 1989 | 0,0 | 0,0 | 0,0 | 0,0 | 0,0 | 0,0 | 0,0 | 0,0 | 0,0 | 0,0 | 0,0 | 55,3 | 69,4 | 175,8 | 326,6 | 565,0 | 759,2 | 14,5 | 30,0 |
| 1990 | 0,0 | 0,0 | 0,0 | 0,0 | 0,0 | 0,0 | 0,0 | 0,0 | 0,0 | 0,0 | 19,2 | 0,0 | 0,0 | 215,1 | 63,6 | 91,1 | 103,6 | 5,4 | 10,6 |
| 1991 | 0,0 | 0,0 | 0,0 | 0,0 | 0,0 | 0,0 | 0,0 | 0,0 | 0,0 | 0,0 | 0,0 | 0,0 | 0,0 | 84,0 | 0,0 | 87,7 | 492,6 | 4,2 | 8,3 |
| 1992 | 0,0 | 0,0 | 0,0 | 0,0 | 0,0 | 0,0 | 0,0 | 0,0 | 0,0 | 13,3 | 0,0 | 50,6 | 64,5 | 82,7 | 476,2 | 260,2 | 845,9 | 14,7 | 28,5 |
| 1993 | 0,0 | 0,0 | 0,0 | 0,0 | 0,0 | 0,0 | 0,0 | 0,0 | 0,0 | 0,0 | 0,0 | 25,3 | 94,9 | 80,3 | 236,0 | 335,3 | 282,5 | 8,6 | 18,1 |
| 1994 | 0,0 | 0,0 | 4,0 | 0,0 | 0,0 | 0,0 | 0,0 | 0,0 | 0,0 | 0,0 | 0,0 | 24,9 | 31,1 | 78,9 | 58,0 | 577,1 | 1111,1 | 13,0 | 23,0 |
| 1995 | 0,0 | 0,0 | 0,0 | 0,0 | 0,0 | 0,0 | 0,0 | 0,0 | 0,0 | 12,9 | 51,3 | 49,0 | 91,7 | 116,4 | 228,2 | 405,2 | 636,9 | 14,7 | 27,4 |
| 1996 | 0,0 | 0,0 | 0,0 | 0,0 | 0,0 | 0,0 | 0,0 | 0,0 | 0,0 | 11,0 | 29,8 | 21,2 | 0,0 | 194,4 | 256,1 | 495,9 | 234,4 | 11,5 | 21,3 |
| 1997 | 0,0 | 0,0 | 0,0 | 0,0 | 0,0 | 0,0 | 0,0 | 0,0 | 0,0 | 0,0 | 0,0 | 0,0 | 57,1 | 76,6 | 101,4 | 488,6 | 461,9 | 8,9 | 16,1 |
| 1998 | 0,0 | 0,0 | 0,0 | 0,0 | 0,0 | 0,0 | 0,0 | 0,0 | 0,0 | 0,0 | 14,5 | 20,6 | 56,4 | 37,8 | 200,2 | 562,7 | 532,3 | 11,2 | 19,9 |
| 1999 | 0,0 | 0,0 | 0,0 | 0,0 | 0,0 | 0,0 | 0,0 | 0,0 | 0,0 | 10,5 | 0,0 | 81,4 | 0,0 | 37,4 | 197,7 | 714,9 | 1352,4 | 17,7 | 29,6 |
| 2000 | 0,0 | 0,0 | 0,0 | 0,0 | 0,0 | 0,0 | 0,0 | 0,0 | 0,0 | 9,4 | 11,8 | 17,2 | 23,5 | 207,3 | 137,0 | 822,5 | 774,7 | 17,1 | 27,7 |
| 2001 | 0,0 | 0,0 | 0,0 | 0,0 | 0,0 | 0,0 | 0,0 | 0,0 | 0,0 | 8,7 | 11,1 | 32,2 | 68,7 | 134,0 | 179,4 | 345,3 | 1030,2 | 15,3 | 26,5 |
| 2002 | 0,0 | 0,0 | 0,0 | 0,0 | 0,0 | 0,0 | 0,0 | 0,0 | 0,0 | 0,0 | 0,0 | 30,0 | 44,0 | 31,7 | 261,7 | 406,2 | 1288,1 | 15,3 | 26,1 |
| 2003 | 0,0 | 0,0 | 0,0 | 0,0 | 0,0 | 0,0 | 0,0 | 0,0 | 0,0 | 0,0 | 30,6 | 14,0 | 0,0 | 30,3 | 296,7 | 531,6 | 797,9 | 13,3 | 22,2 |
| 2004 | 0,0 | 0,0 | 0,0 | 0,0 | 0,0 | 0,0 | 0,0 | 0,0 | 0,0 | 0,0 | 0,0 | 39,5 | 79,9 | 87,3 | 246,5 | 391,4 | 1045,8 | 15,4 | 26,7 |
| 2005 | 0,0 | 0,0 | 0,0 | 0,0 | 0,0 | 0,0 | 0,0 | 0,0 | 6,1 | 0,0 | 0,0 | 12,5 | 94,2 | 28,0 | 238,0 | 383,6 | 1153,1 | 15,1 | 25,6 |
| 2006 | 0,0 | 0,0 | 0,0 | 0,0 | 0,0 | 0,0 | 0,0 | 0,0 | 0,0 | 7,1 | 0,0 | 11,9 | 17,6 | 185,5 | 114,3 | 562,9 | 1505,6 | 17,9 | 30,1 |
| 2007 | 0,0 | 0,0 | 0,0 | 0,0 | 0,0 | 0,0 | 0,0 | 0,0 | 0,0 | 0,0 | 17,3 | 22,7 | 66,0 | 75,7 | 365,8 | 491,4 | 979,8 | 17,2 | 28,7 |
| 2008 | 0,0 | 0,0 | 0,0 | 0,0 | 0,0 | 0,0 | 0,0 | 0,0 | 0,0 | 0,0 | 0,0 | 0,0 | 123,8 | 24,1 | 279,9 | 421,2 | 1253,7 | 16,9 | 28,0 |
| 2009 | 0,0 | 0,0 | 0,0 | 0,0 | 0,0 | 0,0 | 0,0 | 4,9 | 0,0 | 0,0 | 0,0 | 21,0 | 43,8 | 45,8 | 300,9 | 352,5 | 1045,9 | 15,1 | 24,3 |
| 2010 | 0,0 | 0,0 | 0,0 | 0,0 | 0,0 | 0,0 | 0,0 | 0,0 | 0,0 | 0,0 | 7,7 | 30,0 | 26,5 | 140,4 | 351,3 | 652,0 | 901,3 | 22,2 | 29,4 |
| 2011 | 0,0 | 0,0 | 0,0 | 0,0 | 0,0 | 0,0 | 0,0 | 0,0 | 0,0 | 6,0 | 7,3 | 0,0 | 39,4 | 162,3 | 288,7 | 334,6 | 1399,8 | 18,9 | 30,3 |
| 2012 | 0,0 | 0,0 | 0,0 | 0,0 | 0,0 | 0,0 | 0,0 | 0,0 | 0,0 | 0,0 | 7,1 | 9,2 | 0,0 | 37,9 | 31,0 | 542,9 | 1772,9 | 16,5 | 25,6 |
| 2013 | 0,0 | 0,0 | 0,0 | 0,0 | 0,0 | 0,0 | 0,0 | 0,0 | 5,1 | 5,8 | 0,0 | 8,8 | 36,0 | 70,7 | 179,6 | 631,9 | 1093,5 | 16,6 | 25,4 |
| 2014 | 0,0 | 0,0 | 0,0 | 0,0 | 0,0 | 0,0 | 0,0 | 0,0 | 0,0 | 0,0 | 13,3 | 8,5 | 45,7 | 67,1 | 230,1 | 202,6 | 1115,2 | 15,0 | 22,6 |
| 2015 | 0,0 | 0,0 | 0,0 | 0,0 | 0,0 | 0,0 | 0,0 | 0,0 | 0,0 | 0,0 | 12,9 | 8,1 | 21,8 | 159,0 | 166,3 | 635,7 | 1092,0 | 18,5 | 27,2 |
| 2016 | 0 | 0,0 | 0,0 | 0,0 | 0,0 | 0,0 | 0,0 | 0,0 | 0,0 | 0,0 | 19,3 | 24,4 | 32,7 | 79,5 | 221,7 | 391,2 | 1820,1 | 21,9 | 32,2 |
| 2017 | 0,0 | 0,0 | 0,0 | 0,0 | 0,0 | 0,0 | 0,0 | 0,0 | 0,0 | 0,0 | 6,4 | 16,3 | 43,6 | 111,3 | 277,1 | 684,6 | 728,0 | 17,5 | 25,7 |
| 2018 | 0,0 | 0,0 | 0,0 | 0,0 | 0,0 | 0,0 | 0,0 | 0,0 | 0,0 | 0,0 | 0,0 | 40,7 | 43,6 | 63,6 | 138,5 | 391,2 | 936,0 | 14,8 | 21,3 |

| Year | N | | CR | SE | IC95% | |
|---|---|---|---|---|---|---|
| 1980 | 7 | | 5,1 | 1,91122812 | 1,310627189 | 8,802641419 |
| 1981 | 9 | | 6,3 | 2,107437146 | 2,191734632 | 10,45288824 |
| 1982 | 12 | | 8,2 | 2,355522201 | 3,542944748 | 12,77659178 |
| 1983 | 8 | | 5,3 | 1,863430833 | 1,618253881 | 8,922902748 |
| 1984 | 16 | | 10,2 | 2,555975872 | 5,214190778 | 15,23361619 |
| 1985 | 14 | | 8,7 | 2,321242609 | 4,135659041 | 13,23493007 |
| 1986 | 15 | | 9,0 | 2,33527489 | 4,467341973 | 13,62161954 |
| 1987 | 17 | | 10,0 | 2,419237004 | 5,233065173 | 14,71647423 |
| 1988 | 14 | | 8,0 | 2,139067795 | 3,811085938 | 12,19623169 |
| 1989 | 26 | | 14,5 | 2,843895611 | 8,927043817 | 20,07511461 |
| 1990 | 10 | | 5,4 | 1,72271124 | 2,071177238 | 8,824205298 |
| 1991 | 8 | | 4,2 | 1,500666988 | 1,303219917 | 7,185834508 |
| 1992 | 28 | | 14,7 | 2,775724616 | 9,247333837 | 20,12817433 |
| 1993 | 17 | | 8,6 | 2,091131873 | 4,523339119 | 12,72057606 |
| 1994 | 26 | | 13,0 | 2,541618033 | 7,978188601 | 17,94133129 |
| 1995 | 30 | | 14,7 | 2,685085606 | 9,444051765 | 19,96958734 |
| 1996 | 23 | | 11,5 | 2,393499755 | 6,787562055 | 16,17008109 |
| 1997 | 18 | | 8,9 | 2,086024804 | 4,761645092 | 12,93886232 |
| 1998 | 23 | | 11,2 | 2,328898844 | 6,604364758 | 15,73364823 |
| 1999 | 37 | | 17,7 | 2,917504043 | 12,02817635 | 23,4647922 |
| 2000 | 37 | | 17,1 | 2,817567769 | 11,61616283 | 22,66102848 |
| 2001 | 35 | | 15,3 | 2,581693519 | 10,21338554 | 20,33362413 |
| 2002 | 36 | | 15,3 | 2,553191489 | 10,31489362 | 20,32340426 |
| 2003 | 32 | | 13,3 | 2,350275355 | 8,688625434 | 17,90170483 |
| 2004 | 38 | | 15,4 | 2,503701689 | 10,52659844 | 20,34110906 |
| 2005 | 38 | | 15,1 | 2,450309052 | 10,30211369 | 19,90732517 |
| 2006 | 46 | | 17,9 | 2,641227937 | 12,73687267 | 23,09048619 |
| 2007 | 45 | | 17,2 | 2,562143432 | 12,16557952 | 22,20918177 |
| 2008 | 45 | | 16,9 | 2,515205482 | 11,94270856 | 21,80231405 |
| 2009 | 41 | | 15,1 | 2,358859546 | 10,48070602 | 19,72743544 |
| 2010 | 59 | | 22,2 | 2,893261269 | 16,55276941 | 27,89435358 |
| 2011 | 53 | | 18,9 | 2,595395342 | 13,80778843 | 23,98173817 |
| 2012 | 47 | | 16,5 | 2,406877853 | 11,78324263 | 21,21820381 |
| 2013 | 48 | | 16,6 | 2,397045033 | 11,90900688 | 21,30542341 |
| 2014 | 44 | | 15,0 | 2,263351525 | 10,57720656 | 19,44954454 |
| 2015 | 55 | | 18,5 | 2,496944048 | 13,62382234 | 23,41184301 |
| 2016 | 65 | | 21,9 | 2,714464363 | 16,56436119 | 27,20506149 |
| 2017 | 52 | | 17,5 | 2,427890735 | 12,74910323 | 22,26643491 |
| 2018 | 44 | | 14,8 | 2,233334651 | 10,43693022 | 19,19160205 |
