# Supplementary material for: Trends in prostate cancer incidence and mortality to monitor control policies in a northeastern Brazilian state
Source: PLoS One. 2021 Mar 25;16(3):e0249009. doi: 10.1371/journal.pone.0249009 (PMC7993820; doi:10.1371/journal.pone.0249009)
Supplement: S4 Table — (PDF) [file pone.0249009.s004.pdf]

S4 Table. Data quality of incidence data, Aracaju Cancer Registry, 1996-2015.

Basis of diagnosis	TOTAL	1996	1997	1998	1999	2000	2001	2002	2003	2004	2005	2006	2007	2008	2009	2010	2011	2012	2013	2014	2015
Clinical history	5	1														2	2				
Primary site unknown	20	1	1	2	3	2	5	3	1	2											
Histology of metastasis	4														3						1
Histology of primary	3409	36	44	75	103	123	113	136	149	175	215	210	248	225	232	224	227	215	253	198	209
Clinical history + others	21	1	2	3	1	1		1						6		2	1	2		1	
Death certificate only	165	13	5	9	19	12	8	7	5	12	11	7	8	9	6	5	6	7	5	3	8
TOTAL	3624	52	52	89	126	138	126	147	155	189	226	217	256	240	241	233	236	224	258	202	218

	N	%
MV	3413	94,2
DCO	165	4,6
PSU	20	0,6
Total	3624	99,3
