# Supplementary material for: Trends in prostate cancer incidence and mortality to monitor control policies in a northeastern Brazilian state
Source: PLoS One. 2021 Mar 25;16(3):e0249009. doi: 10.1371/journal.pone.0249009 (PMC7993820; doi:10.1371/journal.pone.0249009)
Supplement: S3 Table — (PDF) [file pone.0249009.s003.pdf]

S3 Table. Number of deaths, age-standardized rates and confidence intervals, state area (ST).

	Absolute numbers																		
	Total	Ukn	0	5	10	15	20	25	30	35	40	45	50	55	60	65	70	75	80+
			4	9	14	19	24	29	34	39	44	49	54	59	64	69	74	79	
1980	12													1	1	1	1	4	4
1981	10													1		1	4	1	3
1982	12												1			1	3	2	5
1983	13																3	7	3
1984	21											1			3	4	5	3	5
1985	18												1		1	3	2	4	7
1986	23	2									2	1		1		3	3	6	5
1987	24	2											1	2	4	3	2	6	4
1988	17													1	1	5	3	1	6
1989	28													2	2	4	6	7	7
1990	16	1											1			7	2	1	4
1991	17														2	2		3	10
1992	17														2	2		3	10
1993	23													1	4	3	4	7	4
1994	43	2			1								2	2	2	3	5	10	16
1995	55	4										1	5	4	5	5	9	10	12
1996	42	3										2	2	1	1	8	9	9	7
1997	28										1			1	3	3	4	8	8
1998	34												1	1	3	5	4	7	13
1999	52											1		4	2	5	8	11	21
2000	58											1	1	5	2	6	9	15	19
2001	56											1	1	2	5	5	10	6	26
2002	78													3	4	10	13	12	36
2003	77												4	3	4	5	14	13	34
2004	121											1		5	9	12	16	22	56
2005	139										1		1	4	11	11	18	29	64
2006	143											2		2	6	12	16	32	73
2007	165												3	5	11	14	29	33	70
2008	168										2	1	1	1	15	13	21	34	80
2009	162					1				1				5	16	10	28	29	72
2010	187											1	2	6	6	24	34	31	83
2011	171											1	1	3	15	16	20	27	88
2012	153											1	2	5	9	9	16	35	76
2013	173										1	1	2	7	10	21	19	47	65
2014	186									1		1	3	5	9	15	33	22	97
2015	181												4	1	6	23	24	46	77
2016	157												5	8	9	14	24	30	67
2017	209					1		1				1	3	10	14	19	34	44	82
2018	180													9	8	17	25	31	90

	Age-specific rates																		
	0	5	10	15	20	25	30	35	40	45	50	55	60	65	70	75	80+	Crude	Ag sd
	4	9	14	19	24	29	34	39	44	49	54	59	64	69	74	79		rate	rate
1980	0,0	0,0	0,0	0,0	0,0	0,0	0,0	0,0	0,0	0,0	0,0	7,5	9,6	9,2	13,0	85,4	135,4	2,2	3,4
1981	0,0	0,0	0,0	0,0	0,0	0,0	0,0	0,0	0,0	0,0	0,0	7,6	0,0	9,2	52,1	20,3	92,7	1,8	2,8
1982	0,0	0,0	0,0	0,0	0,0	0,0	0,0	0,0	0,0	0,0	6,3	0,0	0,0	9,1	38,6	39,4	141,0	2,0	3,2
1983	0,0	0,0	0,0	0,0	0,0	0,0	0,0	0,0	0,0	0,0	0,0	0,0	0,0	0,0	38,2	134,0	77,6	2,2	2,9
1984	0,0	0,0	0,0	0,0	0,0	0,0	0,0	0,0	0,0	5,0	0,0	0,0	26,0	36,0	62,8	55,8	119,8	3,4	5,4
1985	0,0	0,0	0,0	0,0	0,0	0,0	0,0	0,0	0,0	0,0	5,8	0,0	8,5	26,8	24,8	72,3	156,0	2,8	4,2
1986	0,0	0,0	0,0	0,0	0,0	0,0	0,0	0,0	7,3	4,6	0,0	6,9	0,0	26,6	36,8	105,5	104,4	3,5	4,6
1987	0,0	0,0	0,0	0,0	0,0	0,0	0,0	0,0	0,0	0,0	5,5	13,6	32,7	26,5	24,3	102,8	78,5	3,6	5,2
1988	0,0	0,0	0,0	0,0	0,0	0,0	0,0	0,0	0,0	0,0	0,0	6,7	8,0	43,8	36,0	16,7	111,2	2,5	3,9
1989	0,0	0,0	0,0	0,0	0,0	0,0	0,0	0,0	0,0	0,0	0,0	13,2	15,8	34,8	71,4	114,3	123,1	4,0	6,0
1990	0,0	0,0	0,0	0,0	0,0	0,0	0,0	0,0	0,0	0,0	5,1	0,0	0,0	60,6	23,5	16,0	67,0	2,2	3,4
1991	0,0	0,0	0,0	0,0	0,0	0,0	0,0	0,0	0,0	0,0	0,0	0,0	15,2	17,2	0,0	46,7	158,9	2,3	3,2
1992	0,0	0,0	0,0	0,0	0,0	0,0	0,0	0,0	0,0	0,0	0,0	0,0	15,2	17,2	0,0	46,7	158,9	2,3	3,2
1993	0,0	0,0	0,0	0,0	0,0	0,0	0,0	0,0	0,0	0,0	0,0	6,2	29,3	24,9	44,9	105,4	61,5	3,0	4,7
1994	0,0	0,0	1,0	0,0	0,0	0,0	0,0	0,0	0,0	0,0	9,4	12,1	14,4	24,5	55,2	148,1	241,5	5,6	7,4
1995	0,0	0,0	0,0	0,0	0,0	0,0	0,0	0,0	0,0	3,7	23,2	23,8	35,4	40,1	97,7	145,5	178,1	7,0	10,1
1996	0,0	0,0	0,0	0,0	0,0	0,0	0,0	0,0	0,0	6,4	8,1	5,3	6,7	66,8	93,4	135,5	90,6	5,3	7,4
1997	0,0	0,0	0,0	0,0	0,0	0,0	0,0	0,0	2,5	0,0	0,0	5,2	19,7	24,6	40,8	118,6	101,9	3,5	4,9
1998	0,0	0,0	0,0	0,0	0,0	0,0	0,0	0,0	0,0	0,0	3,9	5,1	19,5	40,5	40,3	102,4	163,5	4,1	5,9
1999	0,0	0,0	0,0	0,0	0,0	0,0	0,0	0,0	0,0	3,0	0,0	20,3	12,8	39,9	79,5	158,8	261,1	6,2	8,5
2000	0,0	0,0	0,0	0,0	0,0	0,0	0,0	0,0	0,0	2,8	3,3	22,0	11,3	44,6	87,7	200,7	209,3	6,6	8,9
2001	0,0	0,0	0,0	0,0	0,0	0,0	0,0	0,0	0,0	2,7	3,3	8,7	27,7	36,6	96,0	79,1	281,9	6,3	8,4
2002	0,0	0,0	0,0	0,0	0,0	0,0	0,0	0,0	0,0	0,0	0,0	12,8	21,8	72,2	123,0	155,9	385,1	8,6	11,4
2003	0,0	0,0	0,0	0,0	0,0	0,0	0,0	0,0	0,0	0,0	12,7	12,6	21,5	35,6	130,8	166,7	359,1	8,4	10,9
2004	0,0	0,0	0,0	0,0	0,0	0,0	0,0	0,0	0,0	2,6	0,0	20,8	47,8	84,3	147,4	278,8	583,5	13,0	17,0
2005	0,0	0,0	0,0	0,0	0,0	0,0	0,0	0,0	2,0	0,0	3,0	16,1	56,7	75,0	161,1	356,6	648,1	14,4	18,7
2006	0,0	0,0	0,0	0,0	0,0	0,0	0,0	0,0	0,0	5,0	0,0	7,9	30,4	80,6	141,1	387,6	729,1	14,6	18,2
2007	0,0	0,0	0,0	0,0	0,0	0,0	0,0	0,0	0,0	0,0	8,0	16,5	47,1	81,4	236,1	389,6	638,5	16,6	20,4
2008	0,0	0,0	0,0	0,0	0,0	0,0	0,0	0,0	3,5	2,1	2,6	3,3	62,8	74,9	168,8	401,9	729,7	17,2	20,0
2009	0,0	0,0	0,0	1,0	0,0	0,0	0,0	1,5	0,0	0,0	0,0	16,1	64,3	55,8	217,8	336,0	644,8	16,4	19,2
2010	0,0	0,0	0,0	0,0	0,0	0,0	0,0	0,0	0,0	1,8	4,7	17,6	21,8	120,6	239,3	337,5	746,6	18,6	21,2
2011	0,0	0,0	0,0	0,0	0,0	0,0	0,0	0,0	0,0	1,8	2,3	8,7	54,0	79,6	139,5	291,5	784,6	16,8	18,7
2012	0,0	0,0	0,0	0,0	0,0	0,0	0,0	0,0	0,0	1,8	4,6	14,4	32,1	44,4	110,5	374,2	671,7	14,9	16,2
2013	0,0	0,0	0,0	0,0	0,0	0,0	0,0	0,0	1,5	1,6	4,1	18,5	34,2	98,5	136,0	534,5	740,7	16,1	20,9
2014	0,0	0,0	0,0	0,0	0,0	0,0	0,0	1,2	0,0	1,6	5,9	12,7	29,8	67,1	226,4	241,4	1084,9	17,1	22,0
2015	0,0	0,0	0,0	0,0	0,0	0,0	0,0	0,0	0,0	0,0	7,6	2,4	19,1	98,6	157,2	486,3	843,2	16,5	20,6
2016	0	0,0	0,0	0,0	0,0	0,0	0,0	0,0	0,0	0,0	9,1	18,7	27,6	57,7	149,6	305,1	716,3	14,2	17,2
2017	0,0	0,0	0,0	0,0	1,0	0,0	1,0	0,0	0,0	1,5	5,3	22,4	41,4	75,4	201,2	430,2	852,1	18,7	22,2
2018	0,0	0,0	0,0	0,0	0,0	0,0	0,0	0,0	0,0	0,0	0,0	19,3	22,7	65,1	140,4	290,8	906,5	16,0	18,4

Year	N	CR	SE	IC95%		ASR	SE	IC95%	
1980	12	2,2	0,621289967	0,934483242	3,369939911	3,427329742	0,989384874	1,488135388	5,366524095
1981	10	1,8	0,55405362	0,66612629	2,838016482	2,750290306	0,869718159	1,045642714	4,454937898
1982	12	2,0	0,590087304	0,887551266	3,200693497	3,166722826	0,914154138	1,374980716	4,958464937
1983	13	2,2	0,597514401	0,983240585	3,325497038	2,879275023	0,79856721	1,314083291	4,444466755
1984	21	3,4	0,739434005	1,939221649	4,837802947	5,43602829	1,186238625	3,111000586	7,761055995
1985	18	2,8	0,667070332	1,52268188	4,13759758	4,214633208	0,993398574	2,267572003	6,161694413
1986	23	3,5	0,735414862	2,085512649	4,968338908	4,630389743	0,965503004	2,738003855	6,522775632
1987	24	3,6	0,733385652	2,155405386	5,030277142	5,219363055	1,065398022	3,131182931	7,307543179
1988	17	2,5	0,603189173	1,304761894	3,669263453	3,903162546	0,946655968	2,047716849	5,758608243
1989	28	4,0	0,757335426	2,523064958	5,491819828	6,004539021	1,134751213	3,780426643	8,228651399
1990	16	2,2	0,560616678	1,143658024	3,341275403	3,374043385	0,843510846	1,720762126	5,027324643
1991	17	2,3	0,564418536	1,22089691	3,433417571	3,180693681	0,77143153	1,668687882	4,69269948
1992	17	2,3	0,564418536	1,22089691	3,433417571	3,180693681	0,77143153	1,668687882	4,69269948
1993	23	3,0	0,631358604	1,790426632	4,265352359	4,733489325	0,987000753	2,798967849	6,668010802
1994	43	5,6	0,848401384	3,900473206	7,226206631	7,353763893	1,12143848	5,155744472	9,551783314
1995	55	7,0	0,943681977	5,148916177	8,848149529	10,14314714	1,367701681	7,462451843	12,82384243
1996	42	5,3	0,81520084	3,685311613	6,880898904	7,404452147	1,142531771	5,165089876	9,643814418
1997	28	3,5	0,652293488	2,173117467	4,730107942	4,910869848	0,928067167	3,0918582	6,729881495
1998	34	4,1	0,706956002	2,736592675	5,507860202	5,860195994	1,005015322	3,890365964	7,830026025
1999	52	6,2	0,860066976	4,516299892	7,887762438	8,493452694	1,177829969	6,184905955	10,80199943
2000	58	6,6	0,870467582	4,923167142	8,335400064	8,859125132	1,163260119	6,579135298	11,13911497
2001	56	6,3	0,839847096	4,638739872	7,930940488	8,410799038	1,123940298	6,207876053	10,61372202
2002	78	8,6	0,975746094	6,705093827	10,53001852	11,42274713	1,293371424	8,887739135	13,95775512
2003	77	8,4	0,954692907	6,506198164	10,24859436	10,94485748	1,247282268	8,500184239	13,38953073
2004	121	13,0	1,178850985	10,6568129	15,27790876	17,00017199	1,545470181	13,97105044	20,02929354
2005	139	14,4	1,221947509	12,01153154	16,80156577	18,70213244	1,58629417	15,59299587	21,81126902
2006	143	14,6	1,2189657	12,18753691	16,96588245	18,23845742	1,525176429	15,2491162	21,22780322
2007	165	16,6	1,288644342	14,02719338	19,0786792	20,39009789	1,587366968	17,27885863	23,50133715
2008	168	17,2	1,323482014	14,56026275	19,74831225	20,04773497	1,546716332	17,01617096	23,07929898
2009	162	16,4	1,286706652	13,85515694	18,89904702	19,24170641	1,511771232	16,2786348	22,20477803
2010	187	18,6	1,360620545	15,93938985	21,27302238	21,16812155	1,547966356	18,13410749	24,20213561
2011	171	16,8	1,287703835	14,31501314	19,36281218	18,67377004	1,428018886	15,87485302	21,47268705
2012	153	14,9	1,20602136	12,5538585	17,28146223	16,19898714	1,30961049	13,63215058	18,7658237
2013	173	16,1	1,223053195	13,6895689	18,48393743	20,92812132	1,5911356	17,80949554	24,04674709
2014	186	17,1	1,255702209	14,66431856	19,58667122	21,9676202	1,610744063	18,81056184	25,12467857
2015	181	16,5	1,226959578	14,10221212	18,91189366	20,63616677	1,533874196	17,62977334	23,64256019
2016	157	14,2	1,132261139	11,96795957	16,40642324	17,24690019	1,376452484	14,54905332	19,94474706
2017	209	18,7	1,294830318	16,18127734	21,25701218	22,15966776	1,53281627	19,15534787	25,16398765
2018	180	16,0	1,191379268	13,64892681	18,31913354	18,41888183	1,372862395	15,72807153	21,10969212
